# Disruption in the space–time continuum: why digital ethnography matters

**DOI:** 10.1007/s10459-022-10101-1

**Published:** 2022-04-07

**Authors:** Jennifer Cleland, Anna MacLeod

**Affiliations:** 1 Lee Kong Chian School of Medicine, Nanyang Technological University Singapore, Singapore, 308232 Singapore; 2 Division of Medical Education, Dalhousie University, Halifax, Canada

**Keywords:** Digital, Ethnography, Technology, Health professions education research, Sociomateriality

## Abstract

There is increasing interest in the use of ethnography as a qualitative research approach to explore, in depth, issues of culture in health professions education (HPE). Our specific focus in this article is incorporating the digital into ethnography. Digital technologies are pervasively and increasingly shaping the way we interact, behave, think, and communicate as health professions educators and learners. Understanding the contemporary culture(s) of HPE thus means paying attention to what goes on in digital spaces. In this paper, we critically consider some of the potential issues when the field of ethnography exists outside the space time continuum, including the need to engage with theory in research about technology and digital spaces in HPE. After a very brief review of the few HPE studies that have used digital ethnography, we scrutinize what can be gained when ethnography encompasses the digital world, particularly in relation to untangling sociomaterial aspects of HPE. We chart the shifts inherent in conducting ethnographic research within the digital landscape, specifically those related to research field, the role of the researcher and ethical issues. We then use two examples to illustrate possible HPE research questions and potential strategies for using digital ethnography to answer those questions: using digital tools in the conduct of an ethnographic study and how to conduct an ethnography of a digital space. We conclude that acknowledging the pervasiveness of technologies in the design, delivery and experiences of HPE opens up new research questions which can be addressed by embracing the digital in ethnography.

## Introduction

“*All forms of interaction are ethnographically valid, not just the face to face*” (Hine, 2000, p.65).

All of our lives, to some degree, take place in digital spaces. We tweet, we blog, we use Facebook, we text, we WhatsApp, we are “tagged” in something posted by a third party, we are part of online communities. At work, we use email, have remote meetings, upload tasks onto the digital learning management system, use online systems to share data, and so on. Health professions education is increasingly constituted through digitized practices where social interactions and cultural meaning-making processes occur in virtual and online spaces, or in a combination of online and face-to-face spaces (Hine, 2000; Boellstorff et al., 2012; Gatson, 2011).

Digital technology shapes the way we interact, behave, think, and communicate and, in doing so, it has changed the roles of space, place and time in human and material interactions. Space is no longer defined as the congregation of people in any specific place but rather is defined beyond the physical. In other words, space is digitally mediated as well as direct contact with other people (Murthy, 2008). Digital technologies have thus extended the nature of human interactions to encompass “distanced” and dispersed communication and different types of proximity in ways that no other technology has done to date (e.g., Murthy, 2008). Time is fluid and flexible, with asynchronous options for engaging online meaning people participate when it works for them, rather than during mandatory, prescribed periods (Burcks et al., 2019).

If social interactions are no longer solely co-located in time and space, we must extend ethnographic study to settings where interactions are technologically mediated, not just face-to-face (e.g., Bengtsson, 2014; Hine, 2015). Ethnographers who rely principally on face-to-face interviewing and in-person observation are now unlikely to obtain a sufficiently rich picture of their informants’ lives because it is increasingly difficult to separate the online from the embodied, and the digitally-mediated aspects of life now are not amenable to (traditional) observation (Beaulieu, 2010; Czarniawska, 2008).

This ontological shift necessitates thinking differently about ethnographic practices, including the questions that can be addressed, the methods that can be used, and the ethical challenges to consider. What remains the same and what is different when conducting an ethnography of a digital space, compared to “analogue”, or traditional ethnography (Boellstorff et al., 2012; Seligman & Estes, 2020)?

Before discussing this further, it is useful to explain what we mean by digital ethnography. Different authors use different terms to describe their approaches to ethnographic research on digital culture and practices (e.g., ‘digital ethnography’ (Murthy, 2008), ‘virtual ethnography’ (Hine, 2000), ‘cyberethnography’ (Robinson & Schulz, 2009), ‘netnography’ (Kozinets, 2010)) and even different definitions within each of these labels (for example, there are many subtly different definitions of digital ethnography (Hines, 2000, 2008, 2015; Murthy, 2008; Pink et al., 2015)). Common to all these definitions, and the position we take in this paper, is that digital ethnography is research ‘on online practices and communications, and on offline practices shaped by digitalisation’ (Varis, 2016, p.57), and involves human beings studying other human beings (rather than software collecting data: see Kozinets et al., 2018).

Our experience and knowledge of the literature suggests that broadly speaking, the aims of ethnography and digital ethnography are the same: to provide detailed, in-depth descriptions of everyday life and practices (Hammersley & Atkinson, 2007; Hoey, 2014). The practicalities, use of theory and the need for reflexivity are also unchanged. However, the role of the researcher, the notions of field/place and data collection tools differ across traditional and digital ethnography (e.g., da Costa & Condie, 2018; Markham, 2005). Additionally, digital ethnography opens up both new ethical considerations and new opportunities, specifically to examine the complex ways in which culture—the everyday practices, experiences, and understandings of persons interacting digitally—shapes and is shaped by the technological platforms where it occurs (Hine, 2000, 2008).

Despite the fact that bringing the digital into ethnography opens up new vistas of health professions education research (HPER) possibilities, our field has been slow to embrace digital ethnography. A focused search of two mainstream databases identified only six empirical studies which could be considered broadly related to medical education or training (not patient care, or the organisation of care) and which included analysis of some form of digital data (see Box 1). In this era of physical distancing, in which so much of the work of HPE is accomplished online, it is time to foreground digital ethnography.

**Box 1:** Example: Digital ethnography in medical education research

To support our statement that medical education research has yet to embrace digital ethnography in the same way as it has other qualitative methods, we conducted a focused Scopus search on "medical" AND ""ethnography" AND "digital" on 26th March 2021. We set no date limits. This returned 83 papers. We repeated the same search on PubMed. This returned 32 papers. After checking for duplicates we were left with only five empirical studies which could be considered broadly related to medical education or training (not patient care, or the organisation of care) and which included analysis of some form of digital data (Chretien et al., 2015 [Twitter]; Henninger, 2020 [analysis of Reddit discussions]; Macleod & Fournier, 2017 [mobile technology use]; Meeuwissen et al., 2021 [analysis of email communication]); Pérez-Escoda et al., 2020 [healthcare professional use of digital media]). We also specifically searched the Journal of Medical Internet Research using "medical" AND "ethnography" AND "digital". This identified a further 30 papers, one of which met our criteria (Wieringa et al., 2018 [an analysis of physician postings on virtual networks])	

To address this gap and extend understanding of digital ethnography, therefore, we discuss each of three key areas—the use of theory, “new” ethical considerations and digital ethnography practices. We then suggest directions for the development of digital ethnographic studies and best practices in the field of HPE via two detailed examples and other suggestions of how this approach could extend knowledge and understanding in health professions education.

## Theory in digital ethnography

Oversimplified, atheoretical perspectives on the role of technology in research on learning have long characterized the field. Several decades ago, Lave and Wenger noted, “in general, social scientists who concern themselves with learning treat technology as a given and are not analytic about its interrelations with other aspects of a community of practice” (1991, p. 101).

Sociomaterial theoretical perspectives, based on the related concepts of situated learning (Lave & Wenger, 1991), distributed cognition (Hutchins, 1995), and activity theory (Engeström, 1999), offer a robust theoretical starting point for making sense of online activities in the realm of HPE. Now recognized as an important perspective for understanding the active role of digital technologies (MacLeod et al., 2015, 2019), sociomaterial perspectives allow for the theorizing of the entanglement of both social (human) and material (digital) elements—in other words, a sociomaterial assemblage. This, in turn, allows a researcher to explore the social complexities of a digital environment while acknowledging the foundational materiality shaping it.

Orlikowski and Scott (2008) note that the distinction between social and material, or human and technology, is “analytical only, and done with the recognition that these entities necessarily entail each other in practice” (p. 456). Given that technology, the digital environment, and contemporary learning and working practices are inextricably linked, any effort to theorize online HPE activities would be enriched by deliberately attuning to such intra-actions. Well-conceptualised digital ethnographic work should illuminate the ways in which technological elements derive meaning through social agency, and vice-versa. For example, Wesch (2012, p. 101) states that different platforms “create different architectures for participation” and “every feature shapes the possibilities for sociality.” People engage with multiple social media platforms for different purposes, so one sees different content, interactions, and levels of impression management in work emails, WhatsApp, on public platforms like Twitter and more private platforms that may require “friending” someone to see their content (Facebook, Instagram). Different interactions may take place in relatively stable and bounded socio-technical contexts (e.g., discussion forums), compared to more “volatile environments” (Airoldi, 2018 p.662) such as Twitter’s trending topics. Indeed, “specific practices and ways of being human are as likely to differ between online and off, between one form of online activity and another, as between physically located cultures” (Wilson & Peterson, 2002, p 450).

## Ethical considerations

As the amount of human activity on digital media has increased, so too have ethical concerns about doing research within digital spaces. How can a researcher obtain informed consent in digital spaces given the flow of information and flow of users, not all of whom read all messages (Barbosa & Milan, 2019)? What issues need to be negotiated when it comes to friending participants for the purposes of participant observation (Robards, 2013)? These and many other questions have led authors to propose that embracing digital spaces for research purposes challenges “existing conventions of ethics” and requires new ways of approaching such concerns (Markham, 2005; Thompson et al., 2020).

For example, there are different schools of thought about what is public and what is private in the digital sphere (West, 2017). Some researchers posit that messages posted in blogs, chat rooms and any other accessible online forums should be treated as in the public domain and thus do not require informed consent to access. Others argue that just because information is accessible online does not mean participants do not consider it as private or available only to a restricted audience, and therefore informed consent should be required. The concept of contextual integrity (Nissenbaum, 2004) helps here. Briefly, contextual integrity assumes that privacy is related to, and modulated by, the flow of information based on norms that are context-dependant. What context is remains fuzzy, but contextual integrity is based on the notion that individual platforms (e.g., Twitter, Facebook, a workplace) constitute cultural spaces with different practices around privacy and anonymity (Ozkula, 2020).

Second, online practices bring privacy risks (Buglass et al., 2017; Politou et al., 2018). Incidences of “zoom bombings” (e.g., Ling et al., 2021) where people find their way into digital spaces that were intended to be private, including classrooms and PhD defences, are a significant challenge. The increasing sophistication of web search engines mean that quotes that might appear in ethnographic texts can be traced back to the original postings in the public forums, increasing the potential of identification. Researchers may need to paraphrase the participants’ quotations, paying careful attention to changing details of the data, and the social media platform, to assure confidentiality and privacy (e.g., Thompson et al., 2020).

On the other hand, digital communication increases the potential to access, recruit, and engage individuals in one’s research (Caliandro, 2018). Where engagement is purely digital, there are no geographic or social barriers to participation—so hard-to-reach groups may be more accessible (e.g., Morison et al., 2015). Participants may also be more likely to allow a researcher access to sensitive topics where they are already engaging in these discussions online (e.g., the content of a trauma support forum’s online discussion) rather than recalling difficult experiences in a traditional interview. Moreover, social media, messaging apps, and digital tools allow researchers to engage informants/participants in the research process and allow for collaborative ethnography to emerge more easily (Collins & Durington, 2014). Finally, when a data collection period is over, people may keep in touch, providing the researcher with updates via, for example, WhatsApp. These updates can enable follow-up and longitudinal data collection that might not have been possible using more traditional ethnography methods. For example, Robards and Lincoln (2017) in a study of Facebook timelines, or traces, remained friends with their informants after the main data collection period. This allowed them to go back into profiles, revisit particular posts, and clarify events during data analysis.

We also direct readers to Christensen, Larsen and Wind’s (2018) comprehensive guide to the literature on ethical challenges when working with different types of digital data (e.g., social media, online communities, Twitter).

## The practices of digital ethnography

The similarities and differences between analogue and digital ethnography are summarised in Table 1. (Those wishing a deeper dive into the basic tenets of ethnography will find these references helpful [Reeves et al., 2013; MacLeod, 2016; Bressers et al., 2020]). We discuss the field site and the role of the researcher in more detail here.

**Table 1:** Similarities and differences between traditional and digital ethnography

	Traditional ethnography	Digital ethnography
Philosophy	Multiple—e.g., realist, interpretive, critical (e.g., Cuncliffe 2010)	As traditional but also embracing the sociomaterial (e.g., Fenwick et al., 2011)
Aim	To understand culture and social practices and their influences, the “lived reality” of people	
Immersion	Long-term engagement (often months and years) in the field	Anywhere from brief to long term. While immersion in the field may be brief, the nature of digital data and research tools may allow the researcher to trace long-term engagement in a particular topic area using digital footprints
Role of the researcher	Participant observation—interacting with participants/ informants in real life, along the continuum from complete participant to complete observer	Technologically facilitated “being there”
Setting	Traditionally exotic cultures, more recently closer to home (e.g., fieldwork in the community where the researcher lives and works). Referred to as “analogue”	Digital and online environments, or a combination of digital and analogue
The notion of field/place	Situated in time and space	The “place(s)” where the social processes under study occur No time and space boundaries
Data collection tools	Video cameras, voice recorders, kinship diagrams and a notepad	Website archives, blogs, servers, content management systems, bots, laptops, smartphones, tablets, screen shots, chatroom interviews and data
Practicalities	Learning about people, their culture and activities is inherent in all ethnography. Where digital spaces are involved, the researcher must learn to navigate the digital platform or website and learn the etiquette of the online environment—what is acceptable, what is not—to get a sense of situated meanings and the contexts in which they occur. This is no different in principle from any ethnographic immersion	
Reflexivity	The researcher is inherent to the process. The researcher must constantly consider their position in the research and recognise, for example, the implications of their assumptions, previous experiences, etc., on data collection and interpretation	
Theory	Inductive rather than deductive. Accumulation of data and understanding to build toward general patterns or explanatory theories (ie. theory building rather than theory testing)	

### The field site

The concepts of space and a field site are reconceptualised in digital ethnography. The field site is not a single, discrete geographical place but is a “stage on which the social processes under study take place” (Burrell, 2009). This stage may be digital, or it may be part of broader configurations, straddling digital and face-to-face interactions (see Box 2). Examining the entanglement of online and “offline” interactions by “following the thing” (Marcus, 1995) allows fieldworkers to shed new light on the nuanced ways that people engage with different media, how people combine different modes of communication, and the potentially conflicting information that each may yield.

**Box 2:** Communication may be digital or may combine digital and face-to-face interactions

**Online practices** Some social interactions take place solely online. For example, during Covid-19, many groups of students were unable to return to university. While physically displaced from their peers and their places of learning, students could only use digital spaces for communication and social interaction. Addressing questions such as how individuals and groups negotiated a digital presence in light of physical separation, in what ways they used different digital environments during their isolation and how meaningful the online sphere was for social organisation and identity formation is important **Multi-sited practices** Consider a study group. The group may meet weekly to discuss their text(s) of choice. However, members may WhatsApp between meetings to exchange ideas as to the meaning, value and worth of what they have read. They may maintain a Facebook Group where they share other articles about the topic, or even humorous memes about the text. To only observe the meeting would mean many of the group interactions were invisible, whereas being both an observer at the meeting and a member of the WhatsApp or Facebook group would allow the researcher fuller access to the group’s social interactions, and more understanding of their lives	

As always in ethnography, digital ethnographers must determine how to apply boundaries in virtual and other spaces in which they will do their work, but these boundaries are not determined by a physical space. Researchers may decide in advance only to engage with certain content and/or groups, limit their sample size or research question, and provide practical and theoretical reasons for such decisions (Hine, 2015; Markham, 2005). They may also decide to apply boundaries by focusing only on a particular digital platform (see earlier).

### The role of the researcher

Like “traditional” fieldwork, digital ethnographic methods draw on the researcher’s experience as an observer to gain understanding of (digital) culture and communicate the meaning system and practices of its members (Kozinets, 2010). In digital ethnography participation can vary from being identified as a researcher and actively engaging with the participant(s) to covert presence—that is, remaining invisible to the people whose activities are being observed (e.g., Barbosa & Milan, 2019). This lack of visibility—impossible in traditional embodied ethnography where the research is present in space and time—may be an artifact of the challenges of maintaining presence in digital spaces. What we mean by this is that the researcher may disclose their presence, inform participants about the research, and consistently and overtly interact with informants (Murthy, 2008). However, the speed and volume of online activity can mean the researcher fades into the background—participants are often only aware of the people with whom they are directly and actively interacting. On the other hand, “invisibility” may be a conscious decision on the part of the digital ethnographer. This raises the notion of ‘lurking’ (Hine, 2000)—mining for data without interaction, or acting as a participant without researcher transparency. Views on the legitimacy of lurking as a digital ethnographic endeavour vary, from the positive (an opportunity to collect data on unfiltered behaviour), to the wary (can lurking be regarded as ethnographic observation given it is not participatory?) (Varis, 2016), to caution that purely observing interaction increases the risk of misunderstanding the observed (Gold, 1958; Hines, 2000), to considering this unethical research behaviour (e.g., (Doring, 2002). There are also nuances to consider. For example, is it acceptable for a researcher to use covert approaches for a brief period during the planning and early stages of a project, to orient themselves with the digital presence of communities, forms of practice, language, and so on (Hine, 2008)? We cannot offer definitive guidance but raise these issues for consideration.

Researchers need to consider what they bring to the field, and to analysis, in terms of formal, reflexive practices including their personal and social assumptions but also their digital and media persona, and how these may shape the relationship between researcher and participants (Gershon, 2010; Tagg et al., 2017; see also Bourdieu & Wacquant, 1992). Hine (2000) cautions that even the screen name one chooses is an important consideration because it might affect participant engagement. It is also important for researchers to consider their own digital footprint (i.e.,—what information is findable with a Google search), as this may lead to stereotypisation of the researcher, initial distrust or suspicion (Numerato, 2016).

## Applications and opportunities

Given the pervasiveness of digital technologies in the design, delivery and experiences of health professions education, it follows logically that attuning to the digital as an artefact of, and a substrate for, culture will open up new research inquiries and extend knowledge in the field. To illustrate this, we provide two in-depth examples of possible research questions and potential strategies for using digital ethnography to answer those questions. One example looks at how to conduct an ethnography of a digital space and draws on sociomaterial theory. The second example looks at using digital tools in the conduct of an ethnographic study and draws on a theory of social interaction which has been used previously in both traditional and digital ethnographic studies (Kerrigan & Hart, 2016; Leigh et al., 2021) (Boxes 3 and 4).

**Box 3:** Ethnography of a digital space

**Microaggressions experienced by women in medicine: a sociomaterial cyber ethnography** **The case: **Gendered expectations continue to characterize society in general. This is particularly pronounced for women who work in historically male professions, like medicine. Women physicians report experiencing microaggressions daily in their professional roles. **Question: **Why do women physicians turn to Twitter to share stories of the microaggressions they face at work? Role of Theory: Taking a sociomaterial approach purposefully decentres the human to draw attention to other elements of pervasive challenges. In this case, approaching the cyber ethnography from a sociomaterial perspective would allow the researchers to focus on the social media platform “Twitter,” itself, highlighting its opportunities and affordances in building a geographically distributed, like-minded community. **Methods: **Following a traditional ethnographic approach, a cyber ethnography involves the correlation of multiple data sources to build rich description of the issue under study; in this case text, observations, and interview. Distinct from traditional ethnography, however, a cyber approach addresses a field that exists beyond the space/time continuum: online. -Textual Analysis: Search Twitter for the hashtags: #SheMD, #WomeninMedicine, #GirlMedTwitter, #Ilooklikeasurgeon, collecting relevant anecdotes. -Observing and Field noting: “Tweetchat” focused on Women in Medicine (A moderated twitter discussion). -Follow up Interviews: Twitter users who participate in the above hashtags and Tweetchats could be reached by Direct Messaging to invite them to participate in a more formal conversation. Conducting interviews that focus on the affordances of Twitter itself for building a like-minded community (rather than the lived experience of microaggressions). **Analysis: **The data would initially be coded and themed in a traditional ethnographic approach, focusing on description, analysis, and interpretation of each data set as an individual. The next step would be to bring the various coded data sets together to generate a collective story. However, because of the sociomaterial theoretical orientation of the work, the analysis and interpretation would be concentrated on understanding why Twitter, itself, allows for particular types of stories to be shared, and certain types of communities to be developed. This might relate to the ability to be remain relatively anonymous, the ability to connect with colleagues who are living similar experiences but are geographically removed, the appeal of developing “influencer” status, and other such things that are brought about through a network of social and material elements. **Ethical considerations: **The ethical challenges of cyber ethnography mirror those of traditional ethnography. While online activity ostensibly takes place in public, researchers must gauge at what point informed consent is required. **Unique Insights: **Microaggressions facing women in medicine are pervasive. Focusing on how women physicians use Twitter to build a community offers a new angle on a widespread challenge. Rather than asking women physicians to share stories in an interview format, focusing on stories shared and tagged with a particular hashtag, stories generated specifically within the parameters of a “tweet”—generated in real time, with a limited character count, and international reach—provides a very different type of data, and potentially, unexpected insights about how to support women physicians

**Box 4:** Using digital tools to conduct an ethnographic study

**Multiple channels of communication and connectivity** **The case:** Work meetings are increasingly taking place via web-based videoconference platforms such as Zoom or MSTeams. These platforms can be considered the digital frontstage (Goffman, 1959) that structure participant interaction. However, there is a backstage where meeting participants use different digital platforms to interact in simultaneous and asynchronous, spontaneous, and unstructured ways. **Question:** What are the dynamics among online meeting participants, and how do these relate to and influence work outcomes? **Role of Theory:** Taking Goffman’s theories of self-presentation and group dynamics (1959, 1967) allows examination of the relationship between performance and audience, and how different actions may be taken in different regions. In the “frontstage” region—the team-facing Zoom call in this instance—participants must behave in role, consistent with the norms, mores, and laws of the organization and society. While the "official stance" of team members is visible in their frontstage presentation, dissent and deviance may be apparent in the backstage (communications via private chat streams, other digital platforms and in person). **Methods:** Multiple researchers, one taking the role of full participant and one as complete non-participant (Gold, 1958), correlating and interpreting multiple data sources to build rich description of the issue: screen shots and audio recording of the team meeting(s), meeting minutes (text), observations/field notes, and collecting “side conversations” carried out by Whatsapp, text message and emails sent among participants during the meeting. Follow up interviews would also be used, to discuss and reflect on participant communication and presentation in the meeting **Analysis:** The data would be coded and themed in a traditional ethnographic approach. The analysis and interpretation would be concentrated on understanding of how the various digital tools are used in communication, including to control who speaks and who does not, who is muted and so on, and what conversations are going on in parallel (in time, but not in space). **Ethical Considerations:** Ethical consent and informed consent will be collected in advance of data collection. Time will be built into the project to “blend in” and ‘explore the field’, and to meet and inform participants of what the research would entail in the hope that they would then permit us to observe them both front stage and back stage. Any specific concerns (e.g., confidentially issues) will be sought and responded to sensitively. **Unique Insights:** This study offers an opportunity to observe power dynamics, and the subtle acts of resistance that take place back stage, among participants within the work/meetings process. It would also help to develop an understanding of how these in turn influence work outcomes and relationships between team members, potentially giving insights about how to support those with marginalized voices.	

These examples are illustrative. Those wishing to find out more about different approaches to digital ethnography may wish to delve into the empirical references identified in our search and reported in Box 2 (Chretien et al., 2015; Henninger, 2020; Macleod & Fournier, 2017; Meeuwissen et al., 2021; Pérez-Escoda et al., 2020; Wieringa et al., 2018). We also suggest some outstanding research questions and topics which could be explored using various different digital ethnographic practices in Table 2. These suggestions are by no means exhaustive. Rather they reflect our own interests and observations and should be regarded as a springboard to help readers consider diverse ways in which digital ethnography may add knowledge and richness in our field. They also illustrate how the many different approaches encompassed within the broad heading of digital ethnography allow researchers to tailor a methodology according to the research problem.

**Table 2:** A typology of some mainstream digital ethnographic approaches and their actual or potential application to HPER

Approach	Description	What it might bring?	Potential research questions	The question could be explored by	To learn more, read	Examples
Chatnography	Focuses on social media as a communication tool, without making the focus of the work the social media itself	Twitter usage has grown in the last decade; yet, we do not have a good sense of the variety of reasons medical learners choose to connect via this platform	How do medical students construct identity in relation to a particular social issue?	.. observing, and tracking, the patterns of Twitter users related to a relevant “hashtag” (ie. #blacklivesmatter, #metoo	Käihkö (2018)	Chretien et al.’s (2015) digital ethnography of medical students who use Twitter for professional development
Cyber/online/Virtual ethnography	Adapts traditional ethnographic methods (ie. observation) to study communities and cultures generated through computer-medicated communication	In our increasingly digital world, reconfiguring traditional ethnographic methods like observation offers new insights into undertheorized elements of our educational and other practices	How is learner engagement inspired during online continuing professional development offerings?	.. gaining access to web-conferenced events (ie. Zoom, Teams), and taking fieldnotes about various strategies instructors use to include learners	Hine (2008)	Arenas (2019) used virtual ethnography to study online healthcare-seeking behaviours of transgender individuals
Discourse centred online ethnography	Uses systematic observation of online activities and interviews with online actors to be conducted in complement with a linguistic analysis of mobile log data	Ethnographic insights may augment our understanding of how learners use mobile technologies, allowing us to explore technology-mediated discourse, and learn more about learners’ sociolinguistic awareness	How do resident physicians use mobile clinical decision-making software (i.e. UpToDate) in practice?	.. accessing mobile phone logs (with permission) and using automated text mining to understand common queries, followed by interviews with residents focused on why, when, and how they used these tools	Androutsopoulos (2008)	Goergalou (2015) blended online ethnography with discourse analysis, to explore how self-presentation on Facebook is regulated by means of privacy
Netography	Adapted to the unique computer mediated contingencies of today’s social world to learn more about people’s behaviours on the Internet	Understanding how and when people turn to a variety of web-based resources to facilitate their learning would help us learn more about the affordances of various resources	How are study habits influenced by the availability of digital resources?	.. tracking students’ access to a variety of resources, comparing YouTube video hits with ebook downloads from library resources	Kozinets (2015)	Eaton and Pasquini (2020) examined how networked communities of practice (CoP) scaffolded professional learning and development of post-secondary educators
Network ethnography	Mixed method approach using qualitative and quantitative data to study practice (i.e.—everyday sayings and doings)	The HPE community is embracing a practice-turn. Focusing on online behaviour from a practice perspective orients our attention on how everyday activity (in this case, everyday Internet activity) constructs our experiences	How do candidates engage in the practice of preparing for medical school interviews in 2021?	… accessing a variety of internet resources focused on admissions, including Reddit AMAs (ask me anything) about admissions, blogs of students who have successfully gained access, Pinterest boards containing admissions advice, and quantitative metadata of various admissions focused websites	Berthod et al. (2017)	Ball (2016)—looked at educational policy development in India, and how internet spaces change the “geography” or spaces of policy

## Conclusion

Digital ethnography has much to offer in untangling the social and material complexities of health professions education. Bringing the digital into ethnography opens up new research vistas, and allows us to identify and answer questions which are emerge as our practices and interactions become increasingly digitalized. As Hallett and Barber (2014) state, ‘it is no longer imaginable to conduct ethnography without considering online spaces’ (p.307).
